# Mal de Debarquement Syndrome: A Retrospective Online Questionnaire on the Influences of Gonadal Hormones in Relation to Onset and Symptom Fluctuation

**DOI:** 10.3389/fneur.2018.00362

**Published:** 2018-05-24

**Authors:** Viviana Mucci, Josephine M. Canceri, Rachael Brown, Mingjia Dai, Sergei B. Yakushin, Shaun Watson, Angelique Van Ombergen, Yves Jacquemyn, Paul Fahey, Paul H. Van de Heyning, Floris Wuyts, Cherylea J. Browne

**Affiliations:** ^1^Translational Neurosciences, Faculty of Medicine and Health Sciences, University of Antwerp, Antwerp, Belgium; ^2^Department of Otorhinolaryngology and Head and Neck Surgery, Antwerp University Hospital, University of Antwerp, Antwerp, Belgium; ^3^School of Science and Health, Western Sydney University, Sydney, NSW, Australia; ^4^School of Medicine, Western Sydney University, Sydney, NSW, Australia; ^5^Icahn School of Medicine, Mount Sinai Hospital, New York, NY, United States; ^6^Institute of Neurological Sciences, Prince of Wales Hospital, Randwick, NSW, Australia; ^7^Department of Biomedical Physics, Faculty of Sciences, University of Antwerp, Antwerp, Belgium; ^8^Department of Gynaecology, Antwerp University Hospital, University of Antwerp, Antwerp, Belgium; ^9^Translational Neuroscience Facility, School of Medical Sciences, UNSW Sydney, Sydney, NSW, Australia

**Keywords:** Mal de Debarquement syndrome, balance disorder, gonadal hormones, symptom fluctuations, Mal de Debarquement syndrome hormonal profiles, estrogen withdrawal

## Abstract

**Introduction:**

Mal de Debarquement Syndrome (MdDS) is a condition characterized by a persistent perception of self-motion, in most cases triggered from exposure to passive motion (e.g., boat travel, a car ride, flights). Patients whose onset was triggered in this way are categorized as Motion-Triggered (MT) subtype or onset group. However, the same syndrome can occur spontaneously or after non-motion events, such as childbirth, high stress, surgery, etc. Patients who were triggered in this way are categorized as being of the Spontaneous/Other (SO) subtype or onset group. The underlying pathophysiology of MdDS is unknown and there has been some speculation that the two onset groups are separate entities. However, despite the differences in onset between the subtypes, symptoms are parallel and a significant female predominance has been shown. To date, the role of gonadal hormones in MdDS pathophysiology has not been investigated. This study aimed to evaluate the hormonal profile of MdDS patients, the presence of hormonal conditions, the influence of hormones on symptomatology and to assess possible hormonal differences between onset groups. In addition, the prevalence of migraine and motion sickness and their relation to MdDS were assessed.

**Method:**

Retrospective online surveys were performed in 370 MdDS patients from both onset groups. Data were analyzed using Fisher’s exact test or Fisher-Freeman-Hanlon exact test. When possible, data were compared with normative statistical data from the wider literature.

**Results:**

From the data collected, it was evident that naturally cycling female respondents from the MT group were significantly more likely to report an aggravation of MdDS symptoms during menses and mid-cycle (*p* < 0.001). A few preliminary differences between the onset groups were highlighted such as in regular menstrual cycling (*p* = 0.028), reporting menses during onset (*p* < 0.016), and migraine susceptibility after onset (*p* = 0.044).

**Conclusion:**

These results demonstrate a potential relation between hormone fluctuations and symptom aggravation in the MT group. This study is an important first step to suggest a hormonal involvement in the pathophysiology of MdDS and provides a base for further hormonal investigation. Future prospective studies should expand upon these results and explore the implications for treatment.

## Introduction

Mal de Debarquement syndrome (MdDS) has only recently gained greater awareness across the medical and scientific community, though it is still deemed a rare and poorly understood neurological disorder that affects the vestibular system. It is characterized by a chronic perception of self-motion, including rocking, swaying and bobbing. Accompanying symptoms such as brain fog, potential postural instability, mood disorders and migraine have also been described (1, 2). Recently, it has been suggested that MdDS has different subtypes, relating to onset. The more commonly known form of MdDS is defined as Motion-Triggered (MT) MdDS, where symptoms occur after an initial exposure to passive motion (e.g., car ride, boat trip, or flight). However, the same symptomology can arise spontaneously or in the complete absence of a motion event [other onsets, e.g., childbirth, surgery, etc. (3–5)]. These cases are defined as Spontaneous/Other (SO) MdDS. It is still unclear how MT and SO MdDS subtypes overlap or differentiate in clinical aspects, as the literature remains scarce, particularly with regards to the SO MdDS group. However, it has been observed that the two subtypes appear to present alike symptomatically (3). In addition to the limited literature on MdDS and its subtypes, the understanding of MdDS pathophysiology remains unclear. Two main theories have been postulated (4, 5). According to one theory, MdDS has been described as the result of a maladaptive integration of multiple sensory information sources (6). More precisely, it has been hypothesized as a maladaptation of the vestibular ocular reflex, resulting in an altered velocity storage mechanism (7). According to the second theory, MdDS patients present with alterations in neural connectivity, e.g., in the entorhinal cortex and amygdala, with both regions showing hyperactivity. As such, MdDS has been described as a disorder of neuroplasticity (4, 5, 8).

Although many questions remain unanswered, all studies describe a female predominance in MdDS patients (female to male ratio = 9:1). As such, this is considered a typical feature for the condition (1, 2, 4, 5, 8–11). During one of the first clinical assessments of MdDS patients, Hain and Cherchi reported that 80% of the patients included in their study were female (12) and most patients were post-menopausal (11). Although these findings suggest a role for gender, and as such a possible hormonal involvement in MdDS, the potential link between MdDS and gonadal hormones has not yet been investigated. To date, no study has explored the potential role that hormones could play in developing or influencing MdDS.

In general, it is known that females experience hormonal changes throughout their menstrual cycle, reporting mood and behavioral changes in parallel with the fluctuation of hormones, e.g., progesterone, estrogen, and luteinizing hormone (13). Hormonal fluctuations have been found to play an important role in other vestibular disorders, such as vestibular migraine and Ménière’s disease (14). Furthermore, hormonal fluctuations are linked to variations in symptoms of several inner ear disorders, e.g., vertigo, instability, tinnitus, hearing loss, and intra-aural pressure (15, 16). In female vestibular patients, it is suggested that gonadal hormones may have an influence on symptoms, and that specifically, the predominance of vestibular disorders in specific hormonal phases (perimenopause, menopause, etc.) have been overlooked (17). The vestibular apparatus is very sensitive to the influence of pathological and physiological factors (including hormones) that can disturb homeostasis and therefore balance (17).

Another aspect to consider is migraine. Several vestibular pathologies have been shown to be epidemiologically associated with migraine (18), such as Ménière’s disease, benign paroxysmal positional vertigo, psychogenic vertigo and many others. Similarly, MdDS also has a strong interrelation with migraine, with a high number of MdDS patients reporting migraineous symptoms (3, 19). When considering migraine, it is essential to consider hormones. Estrogen and other gonadal hormones have been implicated in migraine symptom fluctuation and pathophysiology (20, 21). From a recent publication (3), it was reported that MdDS patients reported to be affected by migraine after MdDS onset. In migraineous patients, the drop in estrogen, the so called estrogen withdrawal, observed during menses, has been described as the principal cause for migraine vulnerability in females (20). Migraineous female patients also experience symptom variability in response to fluctuating hormonal levels typical of pregnancy, menopause, hormonal replacement therapy (HRT) and the use of hormonal contraceptives (22). Considering hormonal changes throughout different ages, along with migraine headache, dizziness is one of the common symptoms in perimenopause (23). Another condition affected by hormonal fluctuations is motion sickness. Females are known to be generally more prone to motion sickness than males, especially during menses, if not on any form of hormonal contraception (24–26). Interestingly, two-thirds of general migraine sufferers also report to be prone to developing motion sickness (24, 27). As a result, we hypothesize that hormones may be involved in affecting MdDS patients, with a similar mechanism to that of migraine, motion sickness and Ménière’s Disease.

Given the clear predominance of female patients affected by MdDS (1), and acknowledging the influence of gonadal hormones on the nervous system (28) and their roles in more researched vestibular disorders, and other disorders like migraine (20), we aimed to survey female and male MdDS patients within the two onset groups with regards to hormonal profiles, hormonal conditions and symptom fluctuations with regards to different hormonal phases (for females only). Additionally, we aimed to compare the two onset groups where possible. Finally, this study aimed to enquire about their migraine and motion sickness proneness prior to and after MdDS onset, in order to assess if there was a change in migraine and motion sickness susceptibility after MdDS onset.

Our main hypothesis was that female MdDS respondents will report symptom fluctuations in periods that are associated with hormonal changes, particularly phases involving estrogen withdrawal. We hypothesize that the majority of female respondents will report their onset being at the end of their reproductive years, when menopausal transition typically occurs, which could indicate a state of vulnerability for potential MdDS onset. We hypothesize that male and female MdDS respondents will report a higher prevalence of hormonal conditions than what is normally observed in the general population. The hormonally regulated mechanism considered for migraine is here hypothesized as a potential contributor to MdDS pathogenesis.

## Materials and Methods

### Study Population and Recruitment

Patients diagnosed by specialists or believing to suffer from MdDS (also referred to as self-diagnosed patients) were recruited to the study. Inclusion criteria for this study are summarized in Table 1 and are based upon guidelines that have been published earlier (4, 5). Patients were recruited across USA, Europe, and Australia; however, respondents from Asia and South America were also able to access the surveys. MdDS patients were recruited through the Department of Otorhinolaryngology and Head and Neck Surgery (a tertiary referral center) at the University Hospital of Antwerp, Belgium. Patients were also recruited globally through the main MdDS support groups: MdDS Australia Facebook Support Group, MdDS UK Facebook Support Group, website of MdDS Research Group at Mount Sinai Hospital, Western Sydney University MdDS Research Group Facebook page, website and Facebook of Vestibular Disorders Association, website and Facebook of Whirled Foundation and the REACT Community Facebook. Ethical approval was provided by the Ethics Committee of the University Hospital Antwerp Belgium (IRB number 15/44/454) and by the Western Sydney University Human Ethics Committee (H11962). Each respondent provided informed consent. All investigations were conducted according to the principles expressed in the Declaration of Helsinki.

**Table 1:** Inclusion criteria used for this study.

(1)	≥18 years old
(2)	Patients reporting sensations of self-motion (rocking, rocking, swaying and bobbing) for longer than 1 month, where the symptoms could not be explained by another diagnosis
(3)	Patients reporting MdDS symptoms after the exposure to passive motion *(MT group)*
(4)	Patients reporting similar symptoms without a clear motion event or any obvious cause *(spontaneous)*. Patients reporting the initial symptoms after a strong emotional or stressful event (e.g., childbirth, physical or emotional trauma, surgery, etc.) (*other*) (*combined* = *SO group*)

*See guidelines (1, 2, 4)*.

*MdDS, Mal de Debarquement Syndrome; MT, Motion-Triggered; SO, Spontaneous/Other*.

### Questionnaires

The questionnaires were distributed online using two survey platforms; Survey Monkey (MT survey) and Qualtrics (SO survey). The MT survey consisted of 51 questions and the SO survey consisted of 85 questions. More questions were made available to the SO group, as the respondents were re-directed to one of two specific categories: (1) Spontaneous and (2) Other, according to their onset. Additionally, more extensive questions about hormonal profiles were distributed in the SO MdDS survey (questions available as Supplementary Material).

The questions were divided into separate categories in both surveys, from diagnostic to hormonal questions to onset triggers. For more details on the diagnostic and onset features, refer to Mucci et al. (4, 5). With regards to the current manuscript, the categories included are: epidemiology, sensitivity to triggers, hormonal section including: *hormonal fluctuations, imbalances, hormonal clinical conditions and medications* as well as *migraine* and *motion sickness* prevalence reported by MdDS respondents prior and after MdDS onset. Female respondents were required to answer questions about their hormonal status. They were asked if they have reached menopause, defining menopause as 12 months’ absence of the menstrual cycle according to its medical definition (29). The MT and SO surveys were created in two phases. The SO survey was conducted a few months after the MT survey allowing some improvements to be incorporated. For example, in the SO group, the questions relating to menses and mid-cycle/ovulation were distinguished as two separate questions, while in the MT survey those questions were merged in one. In addition to this, at the end of the hormonal section, respondents from both groups and genders were given the opportunity to comment or add information in an open-ended comment section that they believed was relevant to the hormonal section of the survey.

### Normative Data Comparison

As this study lacked a control group, whenever possible, the results from the surveys were compared with normative data, collected from previous publications. The importance of normative data has been previously described (30), so as a result, the most appropriate data that matched age and condition were carefully selected. To increase the quality of the normative data, we considered data from the US population, first because the majority of respondents were US based and second because a larger number of studies were available within the US population only.

### Statistical Analysis

There was little control over sample size and no formal sample size calculations were undertaken prior to data collection. The sample size of 370 was sufficient to provide 95% confidence intervals on proportions with a maximum margin of error of ±5.1% (calculated using: http://www.polarismr.com/help-center/stat-calculator-sample-size/). The per group sample sizes of 266 and 104 provide at least 80% power to detect a difference of 16% points or more between groups (e.g., 50 versus 66%) statistically significant at the 5% significance level (calculated using G*Power Software: http://www.gpower.hhu.de/en.html).

Sample data were summarized using means for age and percentages for the categorical variables. Population results were extrapolated from the sample using 95% confidence intervals. An Independent samples *t*-test for age was used to investigate possible differences between the MT and SO populations. Associations between categorical variables are investigated using Fisher’s exact test (for 2 × 2 tables) or Fisher-Freeman-Hanlon exact test, which are robust to small strata. Due to likely biases in the pattern of missing data, we have included missing observations as a separate category. Warnings about the potential for biases arising from both the volunteer sample and missing responses are included in the discussion. Binomial tests were used to check for any evidence of preference toward a “yes” or “no” answer on questions of the association between menstrual cycle and MdDS symptoms. Statistical analysis was performed with SPSS version 24 (IBM Corp.) and an online calculator for the confidence intervals on proportions was used (online calculator: http://www.sample-size.net/confidence-interval-proportion/).

Responses to open-ended questions were processed by a single coder. When respondents reported extra comments, for example, reflecting their beliefs as to the causes or contributors to their MdDS or to symptom fluctuations, those were tabulated into categories of opinion. Illustrative examples and relative frequencies of particular opinions are provided in the results.

## Results

### Epidemiology—Sample Description

A total of 370 surveys were collected, with 266 (72% of the whole group) being of the MT subtype, and 104 (28% of the whole group) being of the SO subtype. The MT group consisted of 242 females (91% of the MT group), 18 males (6.8%) and 6 respondents did not specify their gender (2.3%). The SO group consisted of 92 females (88.5% of the SO group), 7 males (6.7%) and 5 unspecified (4.8%). The mean age was similar for both groups, i.e., 48.9 years (SD = 11.4) for the MT group and 48.9 years (SD = 13.5) for the SO group. Half of all the respondents from both surveys were from North America (50.9%) and about a quarter each from Europe (25.2%) and Australia (22%). The main characteristics of respondents are summarized in Table 2, where an account of missing data is also provided.

**Table 2:** Demographics and diagnostic confirmation of all respondents, and within the Motion-Triggered (MT) and Spontaneous/Other (SO) onset groups, presented as a percentage of the group and raw number.

				Difference (*p*-value)>
**Age**	**Total ***n*** = 370**	**MT ***n*** = 266**	**SO ***n*** = 104**	
Mean (95% CI)	48.9 (47.7–50.1)	48.9 (47.5–50.3)	48.9 (46.2–51.6)	0.998^a^
SD	12.03	11.4	13.5	
Missing^b^	1.4% (5)	0.4% (1)	3.8% (4)	
**Gender with a missing value category**	**Total ***n*** = 370**	**MT ***n*** = 266**	**SO ***n*** = 104**	
Female (%)	90.3% (334)	91.0% (242)	88.5% (92)	0.476^c^
Male (%)	6.8% (25)	6.8% (18)	6.7% (7)	
Missing	3.0% (11)	2.3% (6)	4.8% (5)	
**Gender excluding missing responses**	**Total ***n*** = 359**	**MT ***n*** = 260**	**SO ***n*** = 99**	
Female (%) (95% CI)	93.0 (89.9–95.4)	93.1 (89.3–95.8)	92.9 (86.0–97.1)	1.000^c^
**Location**	**Total ***n*** = 370**	**MT ***n*** = 266**	**SO ***n*** = 104**	
North America	50.9% (188)	50.9% (135)	51.0% (53)	0.858^c^
Europe	25.2% (93)	25.7% (68)	24.0% (25)	
Australia	22.0% (81)	21.9% (58)	22.1% (23)	
Asia	0.8% (3)	0.8% (2)	1.0% (1)	
South America	1.1% (4)	0.8% (2)	1.9% (2)	
Missing^b^	0.3% (1)	0.4% (1)	0 (0)	
**Officially diagnosed by a health professional a with missing value category**	**Total ***n*** = 370**	**MT ***n*** = 266**	**SO ***n*** = 104**	
Yes	82.4% (305)	86.5% (230)	72.1% (75)	<0.001^c^
No	14.3% (53)	13.5% (36)	16.3% (17)	
Missing	3.2% (12)	0% (0)	11.5% (12)	
**Officially diagnosed by a health professional excluding missing responses**	**Total ***n*** = 358**	**MT ***n*** = 266**	**SO ***n*** = 92**	
Yes (95% CI)	85.2% (305) (81.1–88.7%)	86.5% (230) (81.8–90.3%)	81.5% (75) (72.1–88.9%)	0.306^c^

*The data indicates that officially diagnosed females, from North America in the fifth decade of their lives represented the majority of respondents*.

*^a^Independent samples t-test*.

*^b^Omitted from the hypothesis tested*.

*^c^Fisher’s exact or Fisher-Freeman-Hanlon exact test*.

*CI, confidence interval*.

### Female Hormonal Survey Section

Unfortunately, only around half of the respondents from the SO group completed the hormone-related questions, as some respondents exited the survey before it ended. Data were analyzed including and excluding a missing category. Including the missing category produced statistically significant differences between MT and SO which confirms the potential for response bias in the analyses which excludes the missing data. Results from these questions are summarized in Tables 3–5.

**Table 3:** Hormonal-related data from female respondents in relation to MdDS onset (e.g., menopausal, use of HRT, use of contraceptive and hormonal imbalance conditions), presented as a percentage of the group and raw number.

Females				Difference

				Fisher’s exact test or Fisher-Freeman-Hanlon exact test (*p*-value)
**Menopause including a missing category**	**Total** *n* **= 334**	**MT *n* = 242**	**SO *n* = 92**	
Yes	35.6% (119)	43.0% (104)	16.3% (15)	<0.001
No	50.9% (170)	57.0% (138)	4.8% (32)	
Missing	13.5% (45)	0.0% (0)	48.9% (45)	
**Menopause excluding missing responses**	**Total** *n* **= 289**	**MT *n* = 242**	**SO *n* = 47**	
Yes (95% CI)	41.2% (119)	43.0% (104)	31.9% (15)	0.195
**HRT**	**Total** *n* **= 119**	**MT *n* = 104**	**SO *n* = 15**	
Combined HRT	10.1% (12)	8.7% (9)	20.0% (3)	0.197
Est-only HRT	10.9% (13)	11.5% (12)	6.7% (1)	
No	79.0% (94)	79.8% (83)	73.3 (11)	
**Regular menses including a missing category**	**Total** *n* **= 170**	**MT *n* = 138**	**SO *n* = 32**	
Yes	61.2% (109)	71.0% (98)	34.4% (11)	<0.001
No	29.4% (50)	27.5% (38)	37.5% (12)	
Missing	6.5% (11)	1.4% (2)	28.1% (9)	
**Regular menses excluding missing responses**	**Total** *n* **= 159**	**MT *n* = 136**	**SO *n* = 23**	
Yes	68.6% (109)	72.1% (98)	47.8% (11)	0.028
**PCOS including a not sure and missing category**	**Total** *n* **= 170**	**MT *n* = 138**	**SO *n* = 32**	
Yes	7.1% (12)	7.2% (10)	6.5% (2)	<0.001
No	75.1% (127)	86.2% (119)	25.8% (8)	
Not sure or missing	17.8% (30)	6.5% (9)	67.7% (21)	
**PCOS excluding missing responses**	**Total** *n* **= 140**	**MT *n* = 129**	**SO *n* = 10**	
Yes	8.6% (12)	7.8% (10)	20.0% (2)	0.208
**Hormonal contraceptive use including a missing category**	**Total** *n* **= 170**	**MT *n* = 138**	**SO *n* = 32**	
Combined (Est and Prog)	17.1% (29)	16.7% (23)	18.8% (6)	0.061
Prog only	5.9% (10)	7.2% (10)	0% (0)	
No	74.1% (126)	74.6% (103)	71.9% (23)	
Missing	2.9% (5)	1.4% (2)	9.4% (3)	
**Hormonal contraceptive use excluding missing responses**	**Total** *n* **= 165**	**MT *n* = 136**	**SO *n* = 29**	
Combined (Est and Prog)	17.6% (29)	16.9% (23)	20.7% (6)	0.362
Prog only	6.1% (10)	7.4% (10)	0% (0)	
No	76.4% (126)	75.7% (103)	79.3% (23)	
**Hormonal condition including a not sure and missing category**	**Total** *n* **= 334**	**MT *n* = 242**	**SO *n* = 92**	
Yes (e.g., high test, high Est, HypoT, HyperT, etc.)	15.0% (50)	17.4% (42)/(73% who replied Yes had HypoT)	8.7% (8)/(87.5% who replied Yes had HypoT)	<0.001
No	46.4 (155)	55.0% (133)	23.9% (22)	
Not sure and missing	38.6% (129)	27.7% (67)	67.4% (62)	
**Hormonal condition excluding not sure and missing responses**	**Total** *n* **= 205**	**MT *n* = 175**	**SO *n* = 30**	
Yes	24.4% (50)	24.0% (42)	26.7% (8)	0.818

*Fisher’s exact test is used as well as the Fisher-Freeman-Hanlon exact test was used*.

*MT, Motion-Triggered; SO, Spontaneous/Other; NA, Not Applicable; CI, Confidence Interval, HRT, Hormonal Replacement Therapy; PCOS, Polycystic Ovarian Syndrome; Test, Testosterone; Est, Estrogen; Prog, Progesterone; HypoT, Hypothyroidism; HyperT, Hyperthyroidism*.

**Table 4:** Female respondents who reported specific menstrual phases, and hormonal contraceptive use during the believed onset, presented as a percentage of the group and raw number.

				Difference
				Fisher’s exact test or Fisher-Freeman-Hanlon exact test (*p*-value)
**SO/MT: onset started during menses including a not sure and missing category**	**Total *n* = 170**	**MT *n* = 138**	**SO *n* = 32**
Yes	21.2% (36)	23.9% (33)	9.4% (3)	0.016
No	44.1% (75)	46.4% (64)	34.4% (11)	
Missing and not sure	34.7% (59)	29.7% (41)	56.3% (18)	
**SO/MT: onset started during menses excluding the not sure and missing responses**	**Total *n* = 170**	**MT *n* = 138**	**SO *n* = 32**	
Yes	32.4% (36)	34.0% (33)	21.4% (3)	0.543
**SO/MT: onset started while using a hormonal contraceptive including a not sure and missing category**	**Total *n* = 170**	**MT *n* = 138**	**SO *n* = 32**	
Yes	25.9% (44)	29.7% (41)	9.4% (3)	<0.001
No	56.5% (96)	93 (67.4%)	9.4% (3)	
Not sure and missing	17.6% (30)	2.9% (4)	81.3% (26)	
**SO/MT: onset started while using a hormonal contraceptive excluding the not sure missing responses**	**Total *n* = 170**	**MT *n* = 138**	**SO *n* = 32**	
Yes	31.4% (44)	30.6% (41)	50.0% (3)	0.378
**SO: onset started during ovulation or mid-cycle**	**Total SO *n* = 32**	**Difference (*p*-value)**		
Yes	9.4% (3)			
No	21.9% (7)	NA		
Not sure	62.5% (20)			
Missing	6.3% (2)			

*Fisher’s exact test is used as well as the Fisher-Freeman-Hanlon exact test was used*.

*MT, Motion-Triggered; SO, Spontaneous/Other; NA, Not Applicable*.

**Table 5:** Reported aggravation of symptoms during menses and ovulation, presented as a percentage of the group and raw number.

			Binomial test^^a^^ (*p*-value)
**Symptom aggravation during menses and ovulation including not sure**	**MT *n*** = 149	**MT excluding not sure *n* = 103**	
Yes	49.7% (74)	71.8% (74)	<0.001
No	19.5% (29)	28.2% (29)	
Not sure	30.9% (46)		
**Symptom aggravation during menses including not sure and missing**	**SO *n* = 28**	**SO excluding not sure *n* = 19**	
Yes	39.3% (11)	57.9% (11)	0.648
No	28.6% (8)	42.1% (8)	
Not sure	32.1% (9)		
**Symptom aggravation during ovulation including not sure and missing**	**SO *n* = 28**	**SO excluding not sure *n* = 17**	
Yes	28.6% (8)	52.9% (8)	1.000
No	32.1% (9)	47.1% (9)	
Not sure and missing	39.3% (11)		

*^a^Testing for evidence of a difference in the proportions of yeses and nos*.

*MT, Motion-Triggered; SO, Spontaneous/Other; NA, Not Applicable*.

#### Hormonal Phases, Associated Medications, and Hormonal Conditions

Female respondents from both MT and SO groups were asked if they had reached menopause or were still in an active reproductive phase, more details are reported in Table 3. 41.2% of female respondents (both MT and SO) had experienced menopause, with no statistically significant evidence of any difference between the MT and SO groups (*p* = 0.195), and 58.8% were still in an active reproductive phase. Respondents who answered “yes” to menopause were asked if they had been using HRT, assuming they were in perimenopause or menopause, if they were taking a combined HRT medication (i.e., combined containing both estrogen and progesterone) or an estrogen-only medication. A total of 21% of menopausal respondents were using HRT (combined or estrogen only HRT). The number of respondents from the SO group was small and no statistical significance difference was reached between the two onset groups (*p* = 0.197 Fisher’s exact test). 8.7% of MT subjects and 20% of SO respondents reported to use combined HRT and 11.5% MT and 6.7% SO to use estrogen only HRT, respectively, thus their difference was rather small (*p* = 0.197).

A total of 170 females who reported to be in their active reproductive years (naturally cycling), from both onset groups, were asked whether they had a regular menstrual cycle. The SO group had a much higher rate of missing data than the MT group. After excluding the missing data, the MT group had a statistically significantly higher proportion of females with regular cycles compared to the SO group (72.1 versus 47.8%—Fisher’s exact test *p* = 0.028), suggesting higher menses irregularity within the SO group. About 8.6% of respondents had been diagnosed with polycystic ovarian syndrome (PCOS), with no statistically significant differences between groups when excluding the respondents who did not answer this question or that were unsure. Excluding the less than 3% of missing data, 23.7% of the naturally cycling respondents reported to use hormonal contraceptives, with no statistically significant difference between groups (Fisher-Freeman-Hanlon exact test *p* = 0.362).

Respondents from both groups were asked if they have been diagnosed with any hormonal imbalances or conditions. Definitive answers were provided by 205 respondents. 85% of the female MT respondents but only 14.6% of the female SO respondents answered this question. Excluding the uncertain answers, the majority of both groups reported no hormonal imbalances or conditions. 24.0% of MT and 26.7% of SO reported to have a hormonal condition, respectively, with no statistically significance difference between them (*p* = 0.818). The most reported condition was hypothyroidism in both groups [30 out of 42 (73%) in the MT and 7 out of 8 (87.5%) in the SO group].

#### Menstrual Cycle Phase During Onset

Respondents were asked “to the best of your knowledge, (1) were you menstruating during the motion event that you believe initiated your MdDS? For the SO group only (2) were you ovulating (~2 weeks prior to period) during the time your MdDS symptoms started? and (3) were you on any form of hormonal contraception during the time your MdDS symptoms started?*”* Results are reported in Table 4. Aside from differences in response rates, no further statistically significant differences were observed between MT and SO groups with regards to MdDS onset starting during menstruation (*p* = 0.543) or starting while using hormonal contraceptive (*p* = 0.378) (more details available in Table 4).

#### Menstrual Cycle Phases and Symptom Aggravation

**Figure 1:**
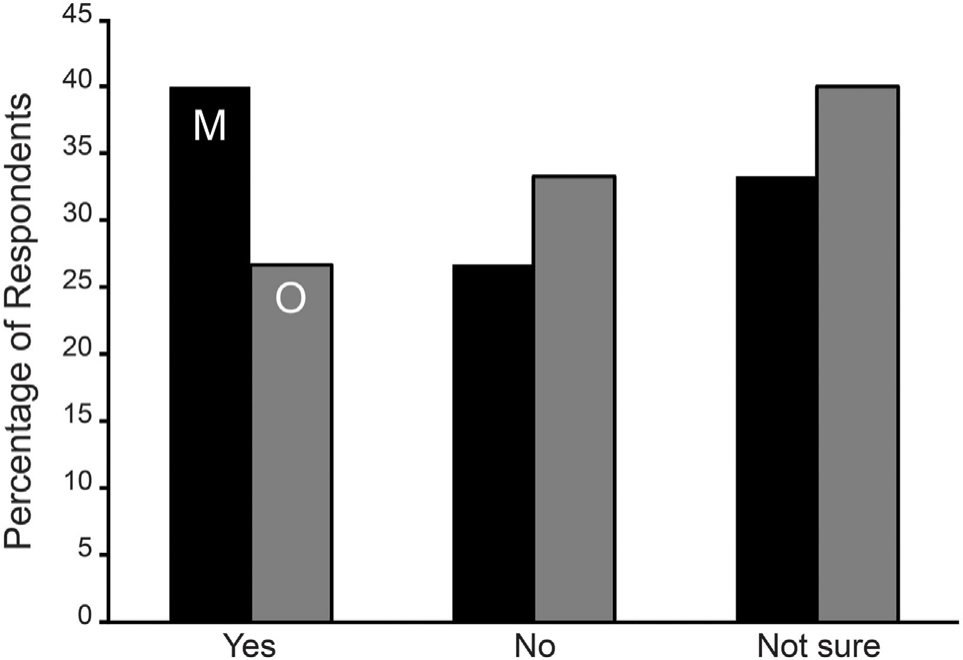
Symptom aggravation during menses (M—black bars) and mid-cycle around ovulation (O—gray bars) in Spontaneous/Other (SO) group expressed as the percentage of respondents. No statistically significant difference was observed among the SO group reporting an aggravation of symptoms during menses or ovulation.

Naturally cycling respondents from both onset groups were asked if their symptoms were normally higher during menses or mid-cycle around ovulation. For the SO group, the two hormonal phases were asked about in two separate questions. We could not compare results between the MT and SO groups. Instead we tested the hypothesis that respondents were more likely to answer this question with a yes rather than no. From Table 5 and Figure 1, it is evident that the MT group were 2.5 times more likely to answer “yes” to an aggravation of symptoms during menses or mid-cycle/ovulation (Binomial test^a^ excluding “not sure” *p* < 0.001), with no corresponding differences in the SO group.

#### Menstrual Cycle Phases and Sensitivity to Triggers

Respondents were asked a series of questions regarding triggers, including “Do you feel that you are more sensitive to your triggers during menses/or during mid-cycle (around ovulation)?” (The SO group had two distinguished questions provided for menstruation and mid-cycle around ovulation). From Table 6, it is evident that the MT group were statistically more likely to say “no” than “yes” to being more sensitive to triggers during menses or mid-cycle around ovulation (*p* < 0.001), while no statistically significant departures from chance variation were found for the SO group.

**Table 6:** Reported sensitivity to triggers during different phases of the menstrual cycle, presented as a percentage of the group and raw number.

			Binomial test^^a^^ (*p*-value)
**More sensitive to triggers during ovulation and menses**	**MT *n* = 148**	**MT excluding not sure *n* = 92**	
Yes	17.5% (26)	28.3% (26)	*p* < 0.001
No	44.5% (66)	71.7% (66)	
Not sure	37.8% (56)		
**More sensitive to triggers during menses**	**SO *n* = 30**	**SO excluding not sure *n* = 19**	
Yes	40% (12)	63.2% (12)	
No	23.2% (7)	36.8% (7)	0.359
Not sure	36.7% (11)		
**More sensitive to triggers during ovulation**	**SO *n* = 30**	**SO excluding not sure *n* = 17**	
Yes	26.7% (8)	47.1% (8)	
No	30% (9)	52.9% (9)	1.000
Not sure	43.3% (13)		

*^a^Testing for evidence of a difference in the proportions of yeses and nos*.

*MT, Motion-Triggered; SO, Spontaneous/Other*.

#### Pregnancy and MdDS

Respondents were questioned about pregnancy during MdDS, with only 6% reporting to have had MdDS while being pregnant. Given that pregnancy is one of the common triggers for SO MdDS, it is not surprising to find that twice the percentage of the SO group were pregnant than the MT group (MT 12/242, 5.0% versus SO 5/45, 11.1%) during onset. However, these numbers were too small to provide evidence of difference from the wider population (*p* = 0.108 one-sided Fisher’s exact test).

### Hormonal Imbalances and Conditions in Males

There were 25 male respondents; 18 (72.0%) in MT and 7 (28.0%) in SO. Both groups were asked if they had been diagnosed with a hormonal imbalance or condition (e.g., low or high testosterone, hypothyroidism). Three out of 18 (16.7%) reported having been diagnosed with low testosterone levels, two SO respondents and one MT respondent, while one SO respondent reported high testosterone. Due to the very low sample size, no significant difference among MT and SO group was reported (*p* = 0.088).

### Overlap of MdDS With Migraine and Motion Sickness

All respondents (male and female) were asked if they experienced migraine and motion sickness before and/or after their MdDS symptoms appeared. A statistical significant difference between the MT and SO groups was observed with regards to migraine, with a higher percentage of SO respondents affected (*p* = 0.044) (Table 7). Similarly, a higher percentage SO respondents (64.0%) reported to be affected by motion sickness after MdDS onset compared to the MT group (45.5%) (Fisher exact test *p* = 0.094). Onset of motion sickness is statistically significantly more likely to occur after MdDS for the SO group (*p* = 0.032) although SO results may be affected by response bias.

**Table 7:** Reported migraine and motion sickness experience with relation to MdDS onset presented as a percentage of the group and raw number.

				Difference
				Fisher’s exact test or Fisher-Freeman-Hanlon exact test (*p*-value)
**Do you experience migraines or frequent headaches/head pressure?**	**Total *n* = 296**	**MT *n* = 264**	**SO *n* = 32**
Yes	67.9% (201)	65.9% (174)	84.4% (27)	0.044
No	32.1% (95)	34.1% (90)	15.6% (5)	
**When did you experience migraines or frequent headaches/head pressure?**	**Total *n* = 201**	**MT *n* = 174**	**SO *n* = 27**	
Before and after MdDS onset	47.3% (95)	47.7% (83)	44.4% (12)	0.063
Before MdDS only	5.0% (10)	3.4% (6)	14.8% (4)	
After MdDS onset only	47.8% (96)	48.9% (85)	40.7% (11)	
**Are you prone to having motion sickness?**	**Total *n* = 289**	**MT *n* = 264**	**SO *n* = 25**	
Yes	47.1% (136)	45.5% (120)	64.0% (16)	0.094
No	52.9% (153)	54.5% (144)	36.0% (9)	
**When were you prone to having motion sickness?**	**Total *n* = 136**	**MT *n* = 120**	**SO *n* = 16**	
Before and after MdDS onset	54.4% (74)	57.5% (69)	31.3% (5)	0.032
Before MdDS only	18.4% (25)	19.2% (23)	12.5% (2)	
After MdDS onset only	27.2% (37)	23.3% (28)	56.3% (9)	

*Fisher’s exact test is used as well as the Fisher-Freeman-Hanlon exact test was used*.

*MdDS, Mal de Debarquement Syndrome; MT, Motion-Triggered; SO, Spontaneous/Other*.

### Normative Data Comparison

Normative data from the literature regarding hormonal characteristics, conditions and medications, and migraine (31–36), taken from the US population, are reported in Table 8.

**Table 8:** Normative data from previous studies of the US population, which correspond to the focal points of this study.

Regular menses in naturally cycling females	Distribution of the regularity and length of menstrual cycles among 4900 females aged 34–45—12% had irregular menses (36)
Polycystic Ovarian Syndrome in naturally cycling females	In a study conducted in 1990, considering Caucasian females, 5.5% of the general female population was estimated to suffer from Polycystic Ovarian Syndrome (33)
Use of hormonal contraceptives in naturally cycling females	25% of females aged 30–35/19.9% of females aged 35–39 (average 22.5%) (34)
Use of hormonal replacement therapy	A study on perimenopausal and menopausal females showed that 20.2% were on some form of hormonal replacement therapy medication (35)
Hypothyroidism	9.4% suffer from hypothyroidism (clinical and subclinical), and this rate increases with age (32)
Migraine	18.9% of females reported severe migraine, twice higher than males (31)

When comparing the data collected from the MdDS respondents (excluding the missing data) with normative data, the number of female MdDS respondents with irregular menses was more than twice as high than the normal population when considering the MT group (27.9 vs. 12%), and was more than fourfold higher in the SO group (52.2 vs. 12%). The percentage of respondents with PCOS was slightly higher (8.6% excluding missing responses) when compared to the normal population (5.5%), considering a 1990 NIH report on White Caucasians (33). Additional normative data reported that 25% of females aged 30–35 years and 19.9% aged 35–39 years reported hormonal contraceptive use (average of 22.5%) (34), which was marginally lower than what was reported in this study (23.7%). 21% of the female respondents who had experienced menopause indicated to be taking either a combined or estrogen-only HRT medication, this is slightly higher than the general population which reports a usage rate of 20.2% in perimenopausal and menopausal females (35). 24.4% (50 of 205) of respondents reported to have some form of hormonal condition, and 37 of these 50 respondents indicated having hypothyroidism (MT and SO together). Therefore, the rate of hypothyroidism in our sample was 10%, which is slightly higher than the prevalence rate of that within the US population, 9.4% (32). The percentage of MdDS respondents who were prone to experiencing migraines was 67.9%, which is significantly higher than the normal population at 18.9% (31).

### Open-Ended Comments

#### Female Respondents

A total of 100 open-ended comments were collected. 74 comments were made by the MT group and 26 from the SO group. Among those, a high number from both onset groups, reported to believe that perimenopause (often expressed by the respondents as *“*going into menopause*”*) or menopause contributed to their MdDS onset (55 out of 100 comments). Similarly, both groups reported to believe that hormones and hormonal imbalances were related to their MdDS symptoms (72 out of 100 comments). A few respondents (13 out of 100 comments), equally from MT and SO groups, reported to have undergone surgeries related to female reproductive organs (e.g., uterine polyp removal, endometrial ablation, hysterectomy or bilateral oophorectomies) in the past and believed that it influenced their MdDS symptoms or pathophysiology. The SO group reported that they believed these surgeries were involved in their initial MdDS onset. Furthermore, for both onset groups, menstruating just before, during or just after being exposed to a motion event or when the spontaneous onset occurred was believed to have influenced or triggered their onset (46% of comments), also ceasing hormonal contraceptive medications was believed to be a trigger for onset in both groups for some respondents (2 out of 100 comments—MT and 2 of out of 100 comments—SO). Additionally, a great proportion of the SO group comments, reported to have higher MdDS symptoms during the week break from their hormonal contraceptive medication. 13 MT and 7 SO respondents reported to have higher symptoms during menstruation, strengthening the previously asked questions. Considering the MT and SO comments together, in total 12 respondents reported to have developed hypothyroidism specifically after MdDS onset. While some respondents specified that while being pregnant (7 out of 24 who indicated that they were pregnant while having MdDS) reported to have had a full remission of MdDS symptoms or a great improvement in symptoms during pregnancy.

#### Male Respondents

A total of seven open-ended comments were collected, five from the MT group and two from the SO group. Two respondents from the MT group and two from the SO group reported to have been taking, using, or experimenting with drugs that influence hormonal levels (e.g., testosterone replacement therapy, anabolic steroids). One respondent believed that his symptoms started during puberty due to the hormonal changes, while another believed his onset was triggered by the combination of steroidal drugs and the use of other recreational drugs.

## Discussion

Given the predominance of female MdDS patients and the lack of investigation into hormonal associations in this condition, a multi-institutional collaboration was setup to collect data from male and female MdDS respondents on a global scale. In total, 370 MdDS patients completed either the MT or SO questionnaire. The current study is the largest, in terms of MdDS respondents recruited to date with regards to hormonal enquiries, and is the only study that compares MT and SO subtypes. Furthermore, this study was the first attempt to link hormonal profiles and clinical hormonal conditions with MdDS onset, symptom fluctuations and other associated features. As the current investigation was based on a retrospective study, we encourage the reader to consider these data as preliminary. The analyzed data suggest a potential relationship does exist between gonadal hormones and the fluctuation and aggravation of MdDS symptoms. From the surveys, a great female predominance was reported as well as an average age within the fifth decade of life (40–50 years old), as expected based upon reports in previous studies (8, 12).

### Hormonal Fluctuations and Aggravation of MdDS Symptoms

**Figure 2:**
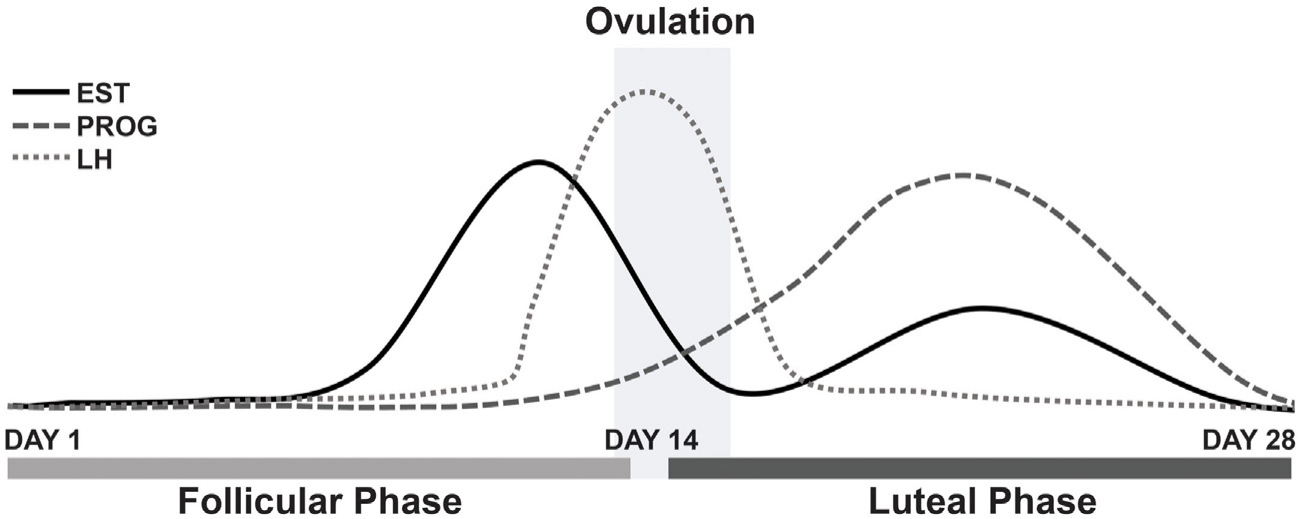
Fluctuations of estrogen, progesterone and luteinizing hormone during a typical 28-day menstrual cycle. Abbreviations: EST, estrogen; PROG, progesterone; LH, luteinizing hormone.

The survey results appear to support our main hypothesis, that estrogen withdrawal is linked to the aggravation of symptoms for female MdDS patients; a majority of which reported an aggravation of symptoms in response to hormonal fluctuations during the menstrual cycle phases. In particular, this aggravation is believed to be caused by estrogen level changes, specifically, by the drop in estrogen. In parallel, this has also been observed in migraine patients during the pre-menstrual phase and menstruation (22). When estrogen is at its lowest, symptoms are heightened (20). The MT group clearly reported an aggravation of symptoms during menstruation and during mid-cycle, around ovulation; when considering the respondents who answered the question only and therefore excluding the missing values. As reported in Figure 2, estrogen rises between days 12 and 14 of the menstrual cycle and then gradually decreases following the luteinizing hormone surge, which is responsible for triggering ovulation (37). Estrogen levels are known to fall around ovulation (mid-cycle), specifically estradiol levels, immediately prior to the luteinizing hormone peak (surge), although is not always observed in all females (38). These significant drops in estrogen may explain the reported aggravation of symptoms around these points in the menstrual cycle.

Similar to the MT results, many respondents from the SO group also reported an aggravation of symptoms linked to hormonal changes. Unlike the MT group, these respondents were asked to distinguish their experience during menses and during mid-cycle around ovulation separately. A large number of SO respondents were unsure as to when their ovulation occurred each month. This posed a great limitation to this question. Consequently, for the SO group, the number of respondents was limited and thus no statistical significance was reached. However, the open-ended comments collected from SO respondents clearly indicated higher MdDS symptoms during menses. Further evaluation of both subtypes with inquiry into defined menstrual cycle phases is encouraged, by perhaps aiding patients through use of commercial urine strips to detect with precision when ovulation is occurring.

In migraine patients, the estrogen withdrawal mechanism is known to be associated with an increase in migraine symptoms and migraine occurrence (39). This has been supported for more than 30 years, since it was proven that the supplementation of estrogen was able to reduce the incidence of migraine attacks until the level of estradiol fell again, however, no reduction in migraine attacks was reported with progesterone (20). A similar improvement of symptoms may be possible for MdDS patients, and thus more research is encouraged to assess if therapeutic hormonal intervention is able to reduce MdDS symptoms.

A specific limitation of these surveys was that questions about premenstrual syndrome (PMS) were not asked, a common condition which affects 30–40% of healthy females in the days prior to menstruation (40), which could be responsible for symptom aggravation in MdDS patients. PMS is characterized by a mix of physical and psychological symptoms (40). In a previous study, it was shown that PMS affects postural stability, where significant increases in postural sway was observed in healthy individuals in the days surrounding menstruation compared to controls (41). As a result, given that healthy females experience changes in postural stability during PMS around menses, it is possible that MdDS patients, who normally experience an increased postural sway, may perceive themselves as even more unstable in the days surrounding menstruation and during menses itself. This phenomenon may be compounded by a natural increase of postural sway during PMS, resulting in an aggravation of symptoms during these times. Future studies should extend upon this by focusing on the potential relationship between MdDS and PMS.

### A Potential Theory on Why Estrogen Fluctuations Could be Related to MdDS Pathophysiology

The influence of the fluctuation of estrogen in MdDS may also lie within the hormone’s ability to affect central nervous system function. Steroid hormones (which include corticosteroids and sex steroid hormones) are able to modulate the physiological and neuroplastic properties of the central nervous system, and affect the existence of specific intracellular steroid receptors in the cerebral cortex, the limbic system, cerebellum and preoptic part of hypothalamus and brain stem (17). For example, changes in estrogen levels may be indirectly related to changes in the optokinetic function (42), as well as inducing changes in the brain functions related to cognitive capacity, memory and mood (43). Another example is provided by assessments on the development of depression, which have been linked to female hormonal function (44).

Estrogen operates *via* two nuclear receptors, estrogen-receptor α and β (45). These receptors operate as transcription factors *via* genomic mechanisms, regulated by an altered expression of target genes (46), as well as by excitatory action within the central nervous system (47). These receptors have been found in brain stem vestibular nuclei concerned with optokinetic, vestibular-ocular and vestibule-spinal reflex (42). This is significant if considering the vestibular ocular reflex maladaptation theory for MdDS (6). Potentially, low levels of estrogen, particularly just after ovulation (known as the luteal phase), may alter the brain’s adaptability to new environments, by influencing the individual’s central neural control for the velocity storage mechanism that has also been implicated in the maladaptation of the vestibular ocular reflex (7). The velocity storage integrative network is constituted of GABA_b_ sensitive neuron receptors. Gamma-aminobutyric acid (GABA) is the main inhibitory neurotransmitter in the human brain and is often the focus of much clinical and neurological research (48). For example, previous studies have shown that low GABA plasma is associated with depression (49) and that low GABA plasma is a characteristic in PMS sufferers in the luteal phase (48). In addition, GABA-mediated neurons are known to have a prominent inhibitory role in spatial navigation. Consequently their deregulation, by altering hormonal levels, could hold some significance in the aggravation of MdDS symptoms (50). If GABA is implicated in MdDS pathophysiology, this could also explain why drugs acting on the release of GABA such as clonazepam may be effective for MdDS patients (10). Evaluating GABA plasma levels in MdDS patients may provide fruitful insights about MdDS pathophysiology. Regarding the second theory, where MdDS is described as a neuroplasticity disorder (3), it might be hypothesized that the hormonal receptors activated by gonadal hormones may also affect the hippocampal and entorhinal cortex interneurons. This suggests that these projections could influence firing patterns within the entorhinal cortex–hippocampal circuit, resulting in symptom changes for MdDS patients. However, more research is needed to evaluate the actual effect of estrogen on MdDS pathophysiology. Additionally, we recognize that for the MT group the question (combining menses and ovulation) was expressed vaguely and a more accurate evaluation should be performed.

### Perimenopause and Menopause in MdDS Patients

Another interesting aspect to consider was the average age of MdDS respondents in both groups (mean age = 48.9 years old SD = 12.03), which matches the fifth decade of life, as previously described (11). This is particularly important in females as it usually indicates a perimenopausal phase (period up to 8 years prior to menopause, characterized with endocrinological, biological and physiological changes), or the menopausal phase (51–53). This supports one of our hypotheses that the majority of female respondents would be within an age range when menopausal transition typically occurs. With regards to menopause, 43% of the females within the MT group reported to be menopausal compared to 16.3% of the SO group, as a result menopausal females were not the major group of respondents engaged in this study. However, great number of respondents from both groups commented that the believed that perimenopause and menopause was involved in triggering their MdDS onset. Fundamentally, this could provide an explanation for the great predominance of female respondents within the same age group, in line with previous research (1, 8, 10, 11). During perimenopause, irregular menstrual cycle length, changes in period pain, or PMS, affect most females (40). Thus, the significant hormonal changes during this phase may lead to an increased period of susceptibility for females; this could be investigated further in prospective surveys and clinical studies. More research of this nature could further elucidate why MdDS is more prevalent in this time frame in women’s lives. In parallel, this is also observed for migraine and dizziness (23), with an equally high incidence in perimenopausal females. In line with our hypothesis, the high number of perimenopausal females may also be linked to estrogen decline, which normally occurs in this phase (20), resulting in potentially altered neurotransmitters and brain regions implicated in MdDS pathophysiology.

### Menstrual Cycle Phases During Onset

Naturally cycling respondents were asked if they were in a particular phase of their menstrual cycle (e.g., menstruation, ovulation) when their MdDS symptoms appeared. The majority of respondents were not sure if they were menstruating or in the middle of their cycle (when ovulation usually occurs) during the time of onset. However, a statistical significant difference was noted between the MT and SO groups when considering the missing variable, with a higher number of MT respondents reporting to have been menstruating during onset. This however could simply indicate a difference in the number of respondents engaging in this question, thus those results should only be considered preliminary. However, to support these data, for both onset groups, a few respondents indicated in the open-ended comment section that they were menstruating just prior, during or soon after the believed onset. From previous investigations, the influence of subtle estrogen fluctuations on brain structural connectivity has been shown in healthy females, in particular regarding the capacity of the human brain to adapt to the environment (54), which has potential relevance for MT patients. This supports our theory that low estrogen levels may be implicated in developing MdDS.

Furthermore, respondents were asked to recall if they were using hormonal contraceptives during the believed onset. A higher number of MT respondents reported to be using hormonal contraceptives compared to the SO group when including the missing data; however, similarly these data should be considered as preliminary. A deeper analysis is needed in order to establish if gonadal hormonal fluctuations are involved in contributing to MdDS onset specifically.

### Triggers and Hormonal Fluctuations

Interestingly, the increase in MdDS symptoms was not directly linked to an increased sensitivity to typical triggers for MdDS (e.g., bright lights, being under stress) during menses. The SO group reported to be more sensitive to triggers during menses compared to the MT group. This could suggest that an increased sensitivity to triggers is not responsible for aggravating MT MdDS symptoms during menses or mid-cycle around ovulation, but rather that the hormonal changes *per se* are responsible for higher MdDS symptoms overall.

### Hormonal Profiles of Female MdDS Respondents

Overall, a difference among the MT and SO groups was reported regarding the regularity of their menstrual cycles. Despite the number of SO respondents being much smaller compared to the MT group, a statistical difference between the two groups was reported, with the SO group reporting to have a higher number of respondents with irregular menses. This could suggest that SO patients may have hormonal imbalances or conditions that makes them more likely to have irregular menstrual cycles and potentially more vulnerable to developing MdDS spontaneously. In addition, both MT and SO groups had a higher incidence rate of irregular menses compared to the general population, which further supports the arguement that MdDS patients may have underlying hormonal issues that make them more susceptible to developing MdDS overall. Another aspect to consider is stress, as high levels of stress are known to affect the regularity of the menstrual cycle, and therefore menses, despite the mechanism remaining unclear (55). Chronic stress induced by persistant MdDS symptoms (4, 5) could be one of the causes for this irregularity. However, this peculiar difference between onset groups should be further examined.

On the contrary, the majority of the MT and SO groups had not been diagnosed with PCOS and, in general, the number of respondents who reported PCOS (excluding missing data) was close to double than that of the normative data [reported rate of PCOS was 5.5% of a female Caucasian population, indicating that this condition can be quite common (33, 56)]. However, a large number of respondents were not sure if they suffered from PCOS (missing and unsure = 6.5% MT; 67.7% SO). Thus, our results should be only considered preliminary. Overall, considering the respondents who confirmed to be suffering from PCOS, it could suggest that MdDS patients have hormonally associated conditions or abnormal hormonal profiles at a higher rate than the general population. Also of interest, a similar number of female respondents from both groups (14.6% MT—12.5% SO) indicated in the open-ended comments that they had undergone hysterectomies or some sort of surgery removing reproductive organs (e.g., uterine polyp removal, endometrial ablation or bilateral oophorectomies). These interventions should be closely evaluated, as it is known they may impact hormonal homeostasis, for example, a recent study reported that after bilateral oophorectomies (surgical removal of the ovaries only), a pronounced and sustained reduction in testosterone levels was recorded in patients (57, 58). Further data are required to confirm a clear relation between hormonal conditions and MdDS.

Respondents were asked whether they had experienced MdDS symptoms whilst being pregnant, and only a very small number responded positively. This can perhaps best be understood as a reflection of the perimenopausal and menopausal hormonal stages that most respondants were in Ref. (11). In addition, some of the respondents who reported to be pregnant while having MdDS symptoms, reported a reduction of symptoms during pregancy, often a full remission of symptoms, which returned shortly after birth. This may reflect the consistently increasing level of estrogen which starts in the first trimester and reaches its peak in the third trimester of pregnancy as well as the absence of cyclic hormonal fluctuations (59, 60). Although anecdotal, it could suggest that MdDS patients might benefit from stable hormonal levels for potential alleviation of MdDS symptoms. This ought to be an important area of future research to further investigate its therapeutic potential. It is also important to note that there is no estrogen withdrawal during pregnancy (and an absence of cyclic fluctuations), but a subsequent withdrawal does occur after delivery. This could suggest a possible mechanism for why some SO patients report childbirth as cause of their onset. An accurate analysis of hormonal changes in respect to MdDS symptoms is needed for pregnant females.

No hormonal clinical dysfunctions were reported overall. However, among the respondents who reported to have a hormone-related condition, hypothyroidism was the most prevalent disorder in both groups. Hypothyroidism, which is characterized by a myriad of symptoms including many that are typically considered as associated symptoms in MdDS as well, such as depression, memory loss, muscle weakness, has been reported to affect 9.4% of the US population (32). The decreased activity of the thyroid leads to many hormonal imbalances and of particular importance to this study, it leads to a decrease in estrogen in females and testosterone in male and female patients (61). Our results indicate a slightly larger number of MdDS sufferers with hypothyroidism (10%) in comparison with the general population. In addition to this, a few respondents reported to have developed hypothyroidism after MdDS onset, in the open-ended comments. Further assessment could be performed in order to evaluate if MdDS patients are unaware of potential thyroid, adrenal or gonadal dysfunctions. Similarly, the incidence of hypothyroidism in respondents affected by Ménière’s disease is high; however, in numerous patients affected by Ménière’s disease, no thyroid enlargment was reported or clinical examination was performed, indicating that patients with less severe thryroid dysfunction may not be diagnosed and therefore improperly managed (62).

### Hormonal Contraceptives, HRT, and MdDS

The number of respondents (from both onset groups) that were taking hormonal contraceptives was small and not significant, although a great majority of SO respondents who contributed to the open-ended comment section, reported to have increased symptoms during the suspension week of contraceptive. This was not the case for the MT group, which in line with a previous epidemiological study no substantial correlation between vestibular symptoms and hormonal contraceptive was found (63). Certain hormonal contraceptives have the ability to restrain the estrogen fluctuation (therefore eliminating estrogen withdrawal), which perhaps holds potential to reduce the symptomatology of some MdDS patients, similar to what has been observed in migraine patients (64). Such treatment includes the continuous use of a combination hormonal contraception, or the use of estrogen alone during the perimenstrual period (64). A further assessment of the SO group is required to confirm why such aggravations occur, as well as if hormonal therapy can influence or improve MdDS symptoms. Furthermore, from the open-ended comment section, four respondents commented that they believed their MdDS onset was triggered after ceasing a hormonal contraceptive medication. Regarding HRT, the number of respondents affected by MdDS and using such medications was small and not relevant for both onset groups. Future studies should assess if hormonal therapies may be relevant (perhaps in a protective role) for MdDS patients.

### Male MdDS Respondents

When considering male respondents, the sample size was relatively small and therefore these results should be considered preliminary. A comparison between the MT and SO group was performed using Fisher’s exact test due to the limited number of SO respondents. A difference among MT and SO group was reported although given the small number of respondents this was not conclusive or significant (*p* = 0.088). Overall, considering the two onset groups together 16.7% reported having been diagnosed with low testosterone levels. Additionally, in the open-ended comments section, it was reported that four out of seven of the male respondents from both groups had reported hormonal imbalances in the past and to be taking a hormonal-related medication (e.g., specifically, some respondents mentioned the use of steroids and recreational drugs). Steroids are known to lead to hypogonadism (65), which causes a wide range of symptoms including loss of libido, erectile dysfunction, diminishing cognitive functions, depression, lethargy, osteoporosis and loss of muscles mass and strength (66). Additionally, published studies conclude that testosterone is an anxiolytic, having a crucial implication in anxiety, exhibiting an antidepressant role as well as improving spatial abilities (57). As a result, testosterone levels could also be explored in male MdDS patients. The results acquired from the male data does not support one of our hypotheses that male respondents would have a higher rate of hormonal conditions compared to the general population, however, given the low response rate from males, this still should be further investigated.

### Migraine and Motion Sickness

When considering migraine and MdDS, migraine proneness featured in both onset groups. Due to the small number of SO (only 32 compared to the 264 respondents from the MT group), Fisher-Freeman-Hanlon exact test was used and a statistical significant difference was present. These results, though preliminary, indicate that a slightly higher number of SO group reported migraine overall, compared to the MT group. Considering both subtypes, from our results it is also clear that an increase in migraine occurrence is associated with having MdDS, and that it is not a predisposing factor, as only a small number of respondents suffered from migraine before onset (3.4% MT; 14.8% SO). The association with migraine has been observed in previous studies (3, 19) especially so for the SO group (19); however, a potential onset difference remains to be elucidated. It has been previously suggested that perhaps MT and SO patients share abnormal brain functioning and physiology, perhaps *via* different pathways or mechanisms (19), and that the mechanism(s) involved in SO MdDS could be closely related with migraine and vestibular migraine. The pathophysiology of vestibular migraine is less understood (67); however, there are several obvious links between central vestibular pathways and proposed mechanisms involved in migraine, which could correlate with MdDS. If considering migraine and dizziness and their potential interaction with ovarian hormones in females, different theories have been proposed. One possible theory applying to both disorders and potentially influencing both onset group relies within the estrogen withdrawal (23).

When considering motion sickness, the number of SO respondents that engaged in this question was much lower than the MT group (264 MT vs. 25 SO), as a result we applied the Fisher-Freeman-Hanlon exact test for this variable. A difference was reported, with a higher number of SO respondents being more susceptible to motion sickness after MdDS onset; however, as reported in the results section, the SO results may be affected by response bias. Overall, there was no evidence to suggest that motion sickness could be a predisposing factor for developing MdDS, given that the majority of MdDS respondents did not report to be particularly prone to motion sickness prior to onset. As previously argued (3), if motion sickness is due to a failure of adaption to passive motion, it would be logical to speculate that people who do not adapt to passive motion would have fewer issues readapting to land. Hain and Cherchi also failed to find a correlation between motion sickness and MdDS, and this may hold some significance for understanding the mechanism(s) involved in each condition (12).

### Study Strengths and Limitations

This is the largest survey performed on people with MdDS and has quite a comprehensive coverage of Western countries. This study is novel in its approach by enquiring about the hormonal aspects of MdDS patients and collating their opinions. It was fully anonymous, allowing respondents to be open. However, we do acknowledge the surveys suffer from non-response and we have no satisfactory method for estimating response rates or response biased in this survey. The best we could do was to clearly present the missing data per enquire and to compare our sample against normative data from the literature, which suggested no major biases in the limited number of variables we examined. The results where a statistically significant difference between the groups was present only when the missing values were included imply that the there is a significant difference in response rates between the two groups. This means there is potential for bias in the following analysis where the missing data are excluded, thus these data should be interpreted carefully.

The survey questions sometimes requested information about events which occurred many years prior and we included no specific strategies for reducing recall bias. The questions were designed for the needs of the current study only and could not be fully validated or tested for reliability prior to use. Further, as this was a volunteer sample rather than a probabilistic sample, the validity of the statistical inference is undermined. All confidence intervals and *p*-values should be interpreted with caution. Access to patients was limited to those active on social media, those who visited webpages related to our studies, or those being assessed at Antwerp University Hospital. As in all retrospective survey studies, our study is also limited by the inability of all respondents to recall or their lack of knowledge regarding specific details, particularly those connecting symptoms patterns to cycle phases of the menstrual cycle (e.g., ovulation). This, as seen in many other surveys (68), made it difficult to assert specific relationships. Also, participant numbers for the SO group were limited. Lastly, a small number of respondents were self-diagnosed, although most patients received a diagnosis from a healthcare professional.

### Future Research

Further research is needed to fully elucidate hormonal involvement and to confirm the hypothesis of estrogen withdrawal being involved in the aggravation of MdDS symptoms. Future studies should consider the implication of estrogen within the vestibular system, for example focusing on estrogen receptors found in brain stem vestibular nuclei, and brain areas concerned with optokinetic, vestibulo-ocular and the vestibulospinal reflex (42), as well as influencing central neurotransmitters (i.e., GABA). Neuroimaging studies, coupled with estrogen testing, could prove useful in assessing the effect of estrogen on hippocampal and entorhinal cortex interneurons, which have been theorized to be involved in MdDS pathophysiology (4, 5). We also encourage future research to conduct a detailed analysis of the perimenopausal phase and PMS in MdDS sufferers; in order to elucidate what other potential causes may be implicated with MdDS symptom fluctuations and onset. The difference in irregular and regular menses for MT and SO subtypes should be further evaluated. Additionally, testosterone testing could prove beneficial for male and female MdDS patients alike to provide detail concerning its effect on cognitive functions and memory (57, 69), which are also described as impaired following MdDS onset. Overall, a clinical assessment of female and male hormonal profiles and underlying hormonal conditions should be performed to elucidate possible mechanisms behind the development of MdDS, and provide direction for potential MdDS hormonal treatment strategies.

## Conclusion

This was the first global survey, with the highest number of MdDS respondents that attempted to identify a link between MdDS and gonadal hormones. From these results, it is evident that hormonal fluctuations are able to influence the symptomology of female MdDS patients (with MT onset), which we hypothesize is due to estrogen withdrawal. Additionally, we have suggested that the hormonal fluctuations occurring in the perimenopausal phase may create a period of vulnerability in females, given the average age of onset of our respondents. We have also suggested that the MdDS population is more likely to have hormonal imbalances or dysfunctions, as supported by our findings of higher rates of PCOS and irregular menstrual cycles, especially in the SO group, when compared to the general population, which may indicate an underlying lack of hormonal homeostasis in our respondents, which potentially contributed to their MdDS onset. Additionally, this study also reported a high prevalence of migraine associated with MdDS and failed to support any significant relationship between motion sickness and MdDS.

This suggests that the mechanism(s) involved in hormonally regulated migraine may be present, or relevant, for female MdDS patients. A theory regarding estrogen influences in MdDS pathophysiology has been proposed, suggesting that more attention should be given to perimenopause and PMS. Although we did not observe a higher rate of hormonal conditions in male respondents when compared to the general population, future studies should consider to examine hypogonadism. Overall, the data revealed that the rate of hypothyroidism in the female MdDS respondents was slightly higher than the general population. Additionally, a few differences between the MT and SO were reported, such as the differences in symptom fluctuation involving menstrual cycle phases, irregular menses, migraine susceptibility, and motion sickness susceptibility.

In conclusion, this study has provided novel insights into the potential hormonal influences within MdDS pathophysiology and peculiar differences related to onset types. These results require future studies to further elucidate the role of hormones in MdDS, and in particular, call for more detailed clinical investigations to help elucidate the role they play in MdDS symptomatology, onset, and finally if hormonal intervention can have a therapeutic potential.

## Ethics Statement

Ethical approval was provided by the Ethics Committee of the University Hospital Antwerp Belgium (IRB number 15/44/454) and by the Western Sydney University Human Ethics Committee (H11962). Each respondent provided informed consent. All investigations were conducted according to the principles expressed in the Declaration of Helsinki.

## Author Contributions

VM is the primary author and was involved in the study design, data analysis, and the writing of the manuscript. JC was involved in the study design and the writing of the manuscript. RB, MD, SY, and SW contributed to the study design and the writing of the manuscript. AO contributed to the writing of the manuscript. YJ contributed to the writing of the manuscript and to the development of the study. PF was involved in the statistics and the writing of the manuscript. PH contributed to the writing of the manuscript. FW provided insights related to data analysis and reviewed this manuscript. CB is the senior author and was involved in designing the entire study, analyzing the results, recruiting respondents, and the writing of the manuscript.

## Conflict of Interest Statement

The authors declare that the research was conducted in the absence of any commercial or financial relationships that could be construed as a potential conflict of interest.

## Abbreviations

GABA, gamma-aminobutyric acid; HRT, hormonal replacement therapy; MdDS, Mal de Debarquement syndrome; MT, Motion-Triggered; PCOS, polycystic ovarian syndrome; PMS, premenstrual syndrome; SO, Spontaneous/Other.
