# Supplementary material for: Mal de Debarquement Syndrome: A Retrospective Online Questionnaire on the Influences of Gonadal Hormones in Relation to Onset and Symptom Fluctuation
Source: Front Neurol. 2018 May 24;9:362. doi: 10.3389/fneur.2018.00362 (PMC5992375; doi:10.3389/fneur.2018.00362)
Supplement: Supplementary file 1 [file presentation_1.PDF]

Supplementary Material

Mal de Debarquement Syndrome: A Retrospective Online Questionnaire on the Influences of Gonadal Hormones in Relation to Onset and Symptom Fluctuation

Viviana Mucci*, Josephine M. Canceri, Rachael Brown, Mingjia Dai, Sergei B. Yakushin, Shaun Watson, Angelique Van Ombergen, Yves Jacquemyn, Paul Fahey, Paul H. Van de Heyning, Floris Wuyts and Cherylea J. Browne*

*** Correspondence:** Corresponding Authors:
Doctor Cherylea Browne: c.browne@westernsydney.edu.au
Doctoral Student Viviana Mucci: viviana.mucci@gmail.com

1 Supplementary Data

Questions analysed and discussed in this manuscript

<u>MOTION TRIGGERED (MT) QUESTIONNAIRE</u>

1. BASIC INFORMATION

1.2: Country/State/City:

1.3: Sex:

[ ] Male
[ ] Female

1.4: Date of Birth:

3. MDDS ONSET AND SYMPTOMS

3.19: Do you experience migraines or frequent headaches/head pressure?

Select one answer:

[ ] Before and after MdDS onset
[ ] Before MdDS only
[ ] After MdDS onset only
[ ]No

3.20: Are you prone to having motion sickness?

Select one answer:

[ ] Before and after MdDS onset
[ ] Before MdDS only
[ ] After MdDS onset only
[ ]No

5. HORMONAL INFLUENCES

FEMALE Q:

5.1: Have you gone through menopause?

[ ] Yes
[ ] No

If yes to menopause

5.2: Are you on Hormone Replacement Therapy (HRT)?

[ ] Yes, combined HRT
[ ] Yes, oestrogen-only HRT
[ ] No

If no to menopause

5.3: To the best of your knowledge, were you menstruating during the motion event that you believe initiated your MdDS?

[ ] Yes
[ ] No
[ ] Not sure

If no to menopause

5.4: Do you feel that your symptoms are worse in the days surrounding menstruation and ovulation (which is usually ~ 2 weeks before your period)?

[ ] Yes
[ ] No
[ ] Not sure

If no to menopause

5.5: Do you feel more sensitive to your triggers in the days surrounding menstruation and ovulation (which is usually ~ 2 weeks before your period)?

Select one answer:

[ ] Yes
[ ] No
[ ] Not sure

If no to menopause

5.6: Are your periods usually regular?

[ ] Yes
[ ] No

If no to menopause

5.7: Are you on any form of hormonal contraception? I.e. Oral contraceptive pill, nuvaring, hormonal patches, implanon)

[ ] Yes, combined Oestrogen + Progesterone hormonal contraception
[ ] Yes, Progesterone only hormonal contraception
[ ] No

If no to menopause

5.8: Were you on any form of hormonal contraception during the motion event that you believe caused your MdDS? I.e. Oral contraceptive pill, nuvaring, hormonal patches, implanon)

[ ] Yes, combined Oestrogen + Progesterone hormonal contraception
[ ] Yes, Progesterone only hormonal contraception
[ ] Yes, but was taking the placebo/sugar pill at that time
[ ] No
[ ] Not sure

5.9: Do you have Polycystic Ovarian Syndrome (PCOS)?

[ ] Yes
[ ] No

5.10: Do you have any hormonal imbalances or conditions?
[ ] Yes, low testosterone
[ ] Yes, high testosterone
[ ] Yes, high oestrogen and progesterone
[ ] yes, high oestrogen only
[ ] yes, high progesterone only
[ ] Hypothyroidism
[ ] Hyperthyroidism
[ ] No
[ ] Not sure

If yes to a condition

5.11: Are you on any medications for your hormonal imbalances or conditions?
[ ] Yes, please specify [free text box]
[ ] No

5.12: Are you pregnant or have you been pregnant whilst having MdDS?
[ ] Yes
[ ] No

MALE Q:

5.13: Do you have any hormonal imbalances or conditions?
[ ] Yes, low testosterone
[ ] Yes, high testosterone
[ ] Yes, high oestrogen and progesterone
[ ] yes, high oestrogen only
[ ] yes, high progesterone only
[ ] Hypothyroidism
[ ] Hyperthyroidism
[ ] No
[ ] Not sure

If yes to a condition

5.14: Are you on any medications for your hormonal imbalances or conditions?
[ ] Yes, testosterone only HRT
[ ] No
[ ] Other [free text box]

FEMALE AND MALE Q:

5.15: Is there anything you would like to add about hormonal influences on your MdDS symptoms or any experience that you feel is appropriate to this section? [free text box]

SPONTANEOUS / OTHER (SO) QUESTIONNAIRE

1. BASIC INFORMATION

1.2: Country/State/City:

1.3: Sex:
[ ] Male
[ ] Female

1.4: Date of Birth:

3. MDDS ONSET AND SYMPTOMS

3.19: Do you experience migraines or frequent headaches/head pressure?

Select one answer:

[ ] Before and after MdDS onset
[ ] Before MdDS only
[ ] After MdDS onset only
[ ]No

3.20: Are you prone to having motion sickness?

Select one answer:

[ ] Before and after MdDS onset
[ ] Before MdDS only
[ ] After MdDS onset only
[ ]No

5. HORMONAL INFLUENCES

Female and Male subjects will be re-directed to different questions. Female subjects will also be directed to different questions according to whether they are on a hormonal contraceptive or not.

FEMALE Q:

5.1: Have you gone through menopause?

[ ] Yes
[ ] No

If yes to menopause

5.2: Are you on Hormone Replacement Therapy (HRT)?

[ ] Yes, combined HRT
[ ] Yes, oestrogen-only HRT
[ ] No
[ ] Other [free text box]

If no to menopause

5.3: To the best of your knowledge, were you menstruating during the motion event that you believe initiated your MdDS?

[ ] Yes
[ ] No
[ ] Not sure

If no to menopause

5.4: To the best of your knowledge, were you ovulating (~ 2 weeks prior to period) during the time your MdDS symptoms started?

[ ] Yes
[ ] No
[ ] Not sure

5.5: Do you have Polycystic Ovarian Syndrome (PCOS)?

[ ] Yes
[ ] No

5.6: Do you have any hormonal imbalances or conditions?

[ ] Yes, low testosterone
[ ] Yes, high testosterone
[ ] Yes, high estrogen and progesterone
[ ] Yes, high estrogen only
[ ] Yes, high progesterone only
[ ] Hypothyroidism

[ ] Hyperthyroidism
[ ] Hypocortisol
[ ] Hypercortisol
[ ] No
[ ] Not sure

5.7: Are you on any medications for your hormonal imbalances or conditions?
[ ] Yes, please specify [free text box]
[ ] No

5.8: Are you pregnant or have you been pregnant whilst having MdDS?
[ ] Yes
[ ] No

5.9: Do you take any hormonal contraceptive? If yes, please specify which one
[ ] Yes [Free text box]
[ ] No

(According to this answer the female subject will be re-directed to two different sections, one for those under hormonal contraceptive and one for those free from hormonal contraceptive).

FEMALE FREE FROM CONTRACEPTIVE:

5.9: Do you feel that your symptoms are worse in the days surrounding menstruation (3 to 4 days before your menstrual cycle)?
[ ] Yes
[ ] No
[ ] Not sure

5.10: Do you feel that your symptoms are worse in the days surrounding ovulation (which is usually ~ 2 weeks before your period)?
[ ] Yes
[ ] No
[ ] Not sure

5.11: Do you feel more sensitive to your triggers in the days surrounding menstruation and ovulation (which is usually ~ 2 weeks before your period)?
Specify if worse during ovulation or menstruation
- Select one answer:

Menstruation:
[ ]Yes
[ ]No
[ ]Not sure

Ovulation:
[ ]Yes
[ ]No
[ ]Not sure

5.12: Are your periods usually regular?
[ ]Yes
[ ]No

5.13: Is there anything you would like to add about hormonal influences on your MdDS symptoms or any experience that you feel is appropriate to this section? [free text box]

FEMALE UNDER CONTRACEPTIVE:

5.14: Specify which hormonal contraceptive you are currently taking? (I.e. Oral contraceptive pill, nuvaring, hormonal patches, implanon)

[ ] Yes, combined Oestrogen + Progesterone hormonal contraception
[ ] Yes, Progesterone only hormonal contraception
Specify the brand and/or name ... [free text box]

5.15: Were you on any form of hormonal contraception during the time your MdDS symptoms started? I.e. Oral contraceptive pill, nuvaring, hormonal patches, implanon)

[ ] Yes, combined Oestrogen + Progesterone hormonal contraception
[ ] Yes, Progesterone only hormonal contraception
[ ] Yes, but was taking the placebo/sugar pill during the suspension/break week at that time
[ ]No
[ ]Not sure

5.16: Do you feel that your symptoms are worse during the days of suspension of the pill?

[ ]Yes
[ ]No
[ ]Not sure

5.17: Do you feel more sensitive to your triggers when off the contraceptive pill?

[ ]Yes
[ ]No
[ ]Not sure

5.18: Is there anything you would like to add about hormonal influences on your MdDS symptoms or any experience that you feel is appropriate to this section? [free text box]

MALE Q:

5.19: Do you have any hormonal imbalances? Select one answer:

[ ] Yes, low testosterone
[ ] Yes, high testosterone
[ ] Yes, high estrogen and progesterone
[ ] Yes, high estrogen only
[ ] Yes, high progesterone only
[ ] Yes, high prolactin
[ ] Hypocortisol
[ ] Hypercortisol
[ ] Andropause
[ ] Hypothyroidism
[ ] Hyperthyroidism
[ ]No
[ ]Not sure

If yes to a condition

5.20: Are you on any medications for your hormonal imbalances or conditions?

[ ] Yes, testosterone only HRT
[ ]No
[ ] Other [free text box]

5.21: Is there anything you would like to add about hormonal influences on your MdDS symptoms or any experience that you feel is appropriate to this section? [free text box]

2 Supplementary Figures and Tables

N/A

2.1 Supplementary Figures

N/A
